# Wilson’s Dental Prosthetics

**Published:** 1914-11-15

**Authors:** 


### BIBLIOGRAPHY.

WILSON'S DENTAL PROSTHETICS. The second edition of this execellent Text Book on Prosthetic Dentistry is just from the press of Lea & Febiger. Like the first edition the author confines his book to plate prosthetics and shows, we think, great wisdom in so doing. He has introduced considerable new material which adds much to the importance of the book. His personal researches on the subjects of plaster of paris as used in dental prosthetics and the vulcanization of rubber are most valuable additions. He has also incorporated a good synopsis of the more recent work of Williams, Gysi, and others on artificial teeth and their articulation. It is our best text book for class room purposes and will also give valuable aid to practitioners.

